# Mapping the network structure of dementia and its associated factors among older adults in Singapore: evidence from two national cross-sectional studies

**DOI:** 10.1186/s12877-026-07535-2

**Published:** 2026-05-13

**Authors:** Ke Ning, Edimansyah Abdin, PV Asharani, Adela-Maria Isvoranu, Sacha Epskamp, Li Ling Ng, Siow Ann Chong, Mythily Subramaniam

**Affiliations:** 1 Research Division, Institute of Mental Health, Buangkok Green Medical Park, 10 Buangkok View, Singapore, 539747 Singapore; 2 Department of Psychology, National University of Singapore, 9 Arts Link, Singapore, 117570 Singapore; 3 Changi General Hospital, 2 Simei Street 3, Singapore, 529889 Singapore

**Keywords:** Dementia, Network analysis, Older adults, Physical activity, Social engagement, Stroke, Sleep health, Singapore

## Abstract

**Background:**

Dementia arises from the interplay of multiple sociodemographic, behavioural, physical, and psychosocial factors that often coexist and interact in later life. Traditional epidemiological studies have typically examined these factors in isolation, overlooking their complex interrelations. Network analysis provides an integrative framework to visualise and quantify these interconnections, offering insights into how dementia and its associated factors coexist within a broader system.

**Methods:**

Data were drawn from two nationally representative cross-sectional surveys of older adults aged ≥60 years in Singapore: the Well-being of the Singapore Elderly (WiSE) 2023 study (*n*=2010) and the WiSE 2013 study (*n*=2565). Dementia was assessed using the 10/66 diagnostic criteria. Variables included factors identified by the Lancet Commission on Dementia Prevention, Intervention, and Care, and established indices including the Lifestyle for BRAin health (LIBRA), Cardiovascular Risk Factors, Aging and Incidence of Dementia (CAIDE), Australian National University Alzheimer's Disease Risk Index (ANU-ADRI), and World Health Organization (WHO) guidelines. Network structure was estimated using mixed graphical models. Sensitivity analyses were conducted to assess the robustness of the network.

**Results:**

Across both surveys, dementia showed the strongest conditional associations with age, educational attainment, employment status, being physically active, walking frequency, stroke, daytime sleepiness, and difficulty maintaining friendships. The overall network structures were comparable in the two surveys, indicating stable interconnections among demographic, behavioural, and social domains. Majority of these interconnections were also not found to vary by gender and ethnicity.

**Conclusions:**

This study highlights the complex web of interrelations linking dementia with social, behavioural, and health-related factors in later life. Rather than implying causal direction, these findings illustrate how multiple factors cluster and coexist within older adults’ lives. These interconnections may inform the design of holistic strategies that integrate employment opportunities, physical activity promotion, social participation, sleep health, and cerebrovascular prevention into comprehensive dementia prevention and care frameworks.

**Supplementary Information:**

The online version contains supplementary material available at 10.1186/s12877-026-07535-2.

## Background

Dementia is a complex and multifactorial syndrome that imposes a major global public health challenge, affecting over 55 million people worldwide, and the burden is expected to triple by 2050 [1]. The extension of the Global Action Plan on the Public Health Response to Dementia from 2025 to 2031 signifies a vital step forward in continuous efforts to deliver "the vision of a world in which dementia is prevented and people with dementia and their carers receive the care and support they need to live a life with meaning and dignity" [2]. Dementia burden is particularly urgent in Asian countries undergoing rapid demographic transitions [3]. Singapore provides a distinctive context for examining dementia and its associated factors. It is one of the fastest ageing societies in Asia, with adults aged 60 years and above comprising 23.0% of the population in 2022 and projected to reach 41.5% by 2050 [4]. In addition, Singapore’s population is multi-ethnic, comprising primarily Chinese, Malay, and Indian ethnic groups, which differ in cultural practices, social structures, and lifestyle patterns that may shape dementia-related risk and protective factors [5].

Existing research has identified a wide range of factors associated with dementia [6, 7], many of which are also implicated in its clinical course and progression [8]. These factors rarely operate in isolation; rather they tend to co-occur and interact within a broader web of interdependencies that shape late-life cognitive health. For example, educational attainment may relate to dementia both directly by building cognitive reserve [9] and indirectly through its influence on health-promoting behaviours [10]. Such behaviours, in turn, are inversely linked to conditions such as obesity, diabetes, and depression, which themselves are mutually reinforcing risk factors [11, 12]. This interwoven structure suggests that dementia risk emerges from complex constellations of sociodemographic, behavioural, physical, and psychosocial elements rather than from single exposures. Traditional unidirectional or linear modelling approaches are limited in capturing these multidimensional interrelations. Adopting a network perspective therefore provides an opportunity to characterise how these diverse domains co-occur and interact with dementia within an interconnected system [13].

Network approaches offer an informative framework for visualising and quantifying the structure of complex interconnections among multiple of factors operating within a system. Unlike traditional models that examine predictors in isolation, network approaches allow numerous variables to be considered simultaneously, thereby capturing the multidimensional nature of ageing-related phenomena. In gerontological research, network analysis has recently been applied to examine conditions characterised by multiple interacting domains, such as frailty [14], and informal dementia care [15]. Within such networks, each variable (node) is connected to others (edges), and those nodes with denser or stronger connections can be identified as more central within the system. These central nodes may represent factors that are more integrally embedded in the overall structure and thus hold greater potential relevance for understanding the broader constellation of associations [16]. Although cross-sectional network analyses cannot establish temporal or causal direction, they offer an exploratory means of characterising the pattern and relative strength of associations among interrelated variables. This perspective can generate hypotheses for subsequent longitudinal and interventional studies aimed at clarifying mechanisms and informing multidomain prevention and care strategies.

While our recent studies have provided updated national estimates of dementia prevalence in Singapore, dementia epidemiology has largely examined associated factors in isolation [17, 18]. This limits understanding of how multiple domains cluster and interrelate in a multi-ethnic, urban ageing population. Network analysis offers a systems-level framework to address this gap by characterizing the structure and interconnections among established dementia-related factors. Leveraging two nationally representative waves of the Well-being of the Singapore Elderly (WiSE) study conducted in 2013 and 2023 with harmonised methodology [17], the present study applies network analysis to examine how diverse factors are interconnected with dementia status, assess the stability of these interrelations over a 10-year period, and investigate whether the pattern or strength of associations differs by gender and ethnicity.

## Methods

### Study population

Two nationwide cross-sectional surveys were conducted in 2013 and 2023 following the same methodology [17, 18]. In each survey, older adults (≥60 years) residing in Singapore, including those living in nursing homes, were randomly selected from an administrative database that comprises the names, ages, and addresses of Singapore citizens and permanent residents using a disproportionate stratified sampling method by age group and ethnicity. Older adults aged 75–84 years and ≥ 85 years, as well as minorities (Malay and Indian), were oversampled to ensure sufficient sample sizes for reliable prevalence estimates across subgroups (Supplementary Table 1). Sampling weights were then calculated to restore the population representativeness of the sample and applied in all analyses (Supplementary Table 1). An informant for each older adult was also recruited to provide additional information, some of which was needed for the 10/66 dementia diagnosis. Detailed information on interviewer training and quality control can be found in previously published papers [17, 18].

### 10/66 dementia diagnosis

Dementia was defined using the 10/66 dementia diagnostic algorithm previously published [19]. It requires:

i) The Geriatric Mental State (GMS) assessment, a semi-structured mental state interview that applies a computer algorithm, the Automated Geriatric Examination of Computer Assisted Taxonomy (AGECAT) [20], for identifying organicity (probable dementia), depression, anxiety, and psychosis.
ii) A cognitive test battery comprising a) the Community Screening Instrument for Dementia (CSI'D) which incorporates the Consortium to Establish a Registry for Alzheimer's Dementia (CERAD) animal naming verbal fluency task and generates the global cognitive score (COGSCORE) [21], and b) the modified CERAD 10-word list learning task with delayed recall [22].
iii) An informant interview using the CSI'D, which generates the informant score (RELSCORE) reflecting the informant's observations of cognitive and functional decline in the respondent [21].

With the above assessment, the 10/66 dementia is defined as those scoring above a cut-off of the predicted probability of the Diagnostic and Statistical Manual of Mental Disorders Fourth Edition (DSM-IV) dementia syndrome [23] from the logistic regression equation developed in the 10/66 international pilot study, which uses coefficients from the GMS, CSI'D (COGSCORE and RELSCORE) and the modified CERAD 10-word list learning task [19]. The 10/66 diagnosis generated by the algorithm was validated in the Singapore population with a clinical diagnosis of dementia using the DSM-IV criteria in our earlier study [18].

### Selection and operationalisation of factors

The selection of factors for network analysis was based on literature and their availability in the dataset. Associated factors of dementia that were mentioned in at least one of the mainstream literature and guidelines, including the 2020 and 2024 reports of the Lancet Commission, popular dementia index- the updated Lifestyle for BRAin health (LIBRA) index, the Cardiovascular Risk Factors, Aging and Incidence of Dementia (CAIDE) index, the Australian National University Alzheimer's Disease Risk Index (ANU-ADRI), and the World Health Organization (WHO) guidelines, were included in the network [6, 7, 24–27]. Operationalisation of all selected variables is illustrated in Supplementary Tables 2&3. For clarity, we categorised the associated factors of dementia into domains of unmodifiable covariates (age, gender, and ethnicity), socioeconomic status (educational attainment); cognitive activity (being employed and highest job level); lifestyle (hazardous drinking, ever smoked, being physically active, any walks of 0.5km or more in the last month, frequency of eating fish, and fruits and vegetables); physical health (hearing impairment, vision impairment, traumatic brain injury (TBI), high blood pressure, diabetes, heart problems, stroke, transient ischemic attacks (TIA), obesity, sleep problems, and daytime sleepiness), mental health (depressive symptoms) and social health (marital status, living alone, frequency of meeting families, friends, and neighbours, number of friends and neighbours, attending religious activities and community meetings or social clubs, feeling lonely, satisfaction with friend support in neighbourhood, and difficulty in maintaining friendship).

Categorical and continuous variables were dichotomised where applicable to improve the visualization of the network structure as mixed graphical modelling (MGM) does not define sign for unordered categorical variable and cannot deal with ordered categorical variables (Supplementary Table 1) [28]. Dichotomisation of variables was informed by established epidemiological conventions or clinical relevance [29] while accounting for their distributional properties. For example, age was dichotomised as ≥75 years or below 75 years, which is commonly used in dementia research to distinguish older-old population at higher risk [30, 31]. When appropriate cut-points could not be determined, variables were instead recoded as unordered categorical variable (i.e. ethnicity, frequency of consuming fish and fruits and vegetables, number of friends and neighbours one meets regularly) (Supplementary Table 2).

### Statistical analyses

Our primary analyses were to construct a network structure among dementia and its associated factors using WiSE 2023 study, and then replication analyses were conducted using WiSE 2013 study. To ensure generalisability of the findings to the national population, sampling weights were calculated using census population distribution (June 2022 for WiSE 2023 and June 2011 for WiSE 2013) and were applied throughout all analyses. The accuracy and stability of the estimated network were checked, and a series of sensitivity analyses were conducted. All the analyses were carried out in R version 4.2.3. Supplementary Table 1 illustrates the procedures of all analyses and key considerations.

A mixed graphical model (MGM) was conducted using the mgm package (version 1.2–14.2) to incorporate the mix of binary and unordered categorical variables under investigation [28]. Cross-validation was used to select the tuning parameter controlling the L1-penalization instead of Extended Bayesian Information Criterion (EBIC) as the latter produces unreasonably sparse network (Supplementary Figure 1). Meanwhile, as the CV tends to be less conservative than the EBIC [28], to avoid false positive results, we only presented edges with a weight ≥0.22 for clarity (exponentiate of ±0.22 is 0.8/1.25, which is approximately the minimal clinically important differences utilised by the guidelines for the National Institute for Health and Clinical Excellence (NICE) in the UK [32].

After network estimation, shortest paths between each factor and dementia (defined as the minimum number of steps needed to go from one node to the other) were computed using Dijkstra's algorithm to identify potential pathways [33]. Because all variables included in the network were dichotomous or unordered categorical and thus measured on comparable scales, node strength was computed as the sum of absolute edge weights connected to a given node and used as a within-network, rank-based measure without additional normalisation [34]. The accuracy of the network was examined by 1) drawing nonparametric bootstrapped confidence intervals of the edge-weights, 2) conducting the case-dropping subset bootstrap to calculate the correlation-stability coefficient (the maximum proportion of cases that can be dropped to retain, with 95% certainty, a correlation with the original centrality of higher than 0.7) of node strength, and 3) testing for significant differences between edge-weights of interest by applying bootstrapped difference tests [35].

Comparison of network structure of WiSE 2013 and of WiSE 2023 was examined by calculating edge-weight correlation between two networks and conducting the moderated network analysis [36]. This approach identifies differences in network structure across subgroups, hence was also implemented to investigate whether each pairwise association between variables in the network varied by gender and ethnicity, offering insights into whether universal or tailored interventions would be more effective.

All the models were visualised using the *qgraph* package (version 1.9.8), with ‘nodes’ representing variables and ‘edges’ representing the conditional dependency between the variables. Signs of association for binary variables are defined as positive (blue) or negative (red) but are undefined (grey) for categorical variables.

#### Missing data

The network comprised a large set of dementia-related determinants (42 nodes), resulting in only 1,140 of 1,993 participants with complete data on all variables. A complete-case network analysis was therefore not pursued, as it would substantially reduce sample size and compromise population representativeness given the use of sampling weights and notable differences in the distributions of variables between complete cases and noncomplete cases (Supplementary Table 4). Instead, multiple imputation was applied. Although 42.8% of participants had at least one missing value, missingness for individual variables was generally low, except for obesity status (30.9%) and dementia diagnosis (10.5%, due to the unavailability of informants required to provide the necessary information for the 10/66 dementia diagnosis) (Supplementary Table 4), and only 8.8% had three or more variables missing (Supplementary Table 5). Nonetheless, sensitivity analyses excluding high-missingness variables and participants were conducted to assess potential imputation-induced bias (see below). Multiple imputation (MI) was conducted using the *mice* package (version 3.16.0) to account for missing values, and 20 datasets were imputed [37]. More details are available in Supplementary Table 1 & Text 1.

#### Sensitivity analysis

First, variables in the network were recoded as unordered categorical variables to reserve more information about the variables. Currently, the *mgm* package for conducting mixed graphical modelling does not define signs for unordered categorical variables; thus, for a better visualisation of a network of categorical variables in this sensitivity analysis, we defined signs among categorical variables as detailed in Supplementary Text 2. Second, emerging associated factors of dementia, including personal income, household wealth, number of life deficits, tooth loss, and anxiety, were added to the network to assess robustness of the network. Third, factors that had disproportionately high percentages of missing values among people with and without dementia, including obesity, daytime sleepiness, feeling lonely and satisfaction with friend support in the neighbourhood, were omitted from the network. Fourth, variables with direct and strong associations with dementia that were also highly correlated with other variables (>0.8) were omitted from the network to avoid random selection of highly correlated variables by LASSO [38]. Fifth, the "multiple imputation, then deletion" (MID) method was implemented, deleting participants with imputed diagnoses of dementia during analysis [39]. Sixth, we excluded participants whose information on sociodemographic and risk factors was supplemented by informants (53.6% of participants diagnosed with dementia, compared with 1.6% of those without dementia).

## Results

After excluding participants identified as “Others” in ethnicity, 1993 older adults from WiSE 2023 were included in the analysis, with 678 Chinese, 699 Malay, and 616 Indian participants. Of these, 1784 participants had informants and therefore could be assessed for dementia. Unweighted and weighted mean age of the older population was 74.2 (standard deviation: 9.2) and 70.8 (standard deviation: 7.7), respectively. People diagnosed with dementia were different from those undiagnosed in various aspects (Table 1).

**Table 1:** Distribution of associated factors by diagnosis of dementia in WiSE 2023

**Variable labels**	**level**	**Non-dementia** **(*n*=1476, 82.7%)**	**Dementia** **(*n*=308, 17.3%)**
Unmodifiable covariates			
Age	60–74	929(62.9%)	34(11.0%)
	75 and above	547(37.1%)	274(89.0%)
Gender	Male	690(46.7%)	110(35.7%)
	Female	786(53.3%)	198(64.3%)
Ethnicity	Chinese	516(35.0%)	66(21.4%)
	Malay	515(34.9%)	131(42.5%)
	Indian	445(30.1%)	111(36.0%)
Socio-economic status			
Educational attainment	Primary or below	820(55.6%)	245(80.3%)
	Secondary or above	655(44.4%)	60(19.7%)
Cognitive activity			
Being employed	No	928(62.9%)	297(97.1%)
	Yes	548(37.1%)	9(2.9%)
Highest job level	Manager/professional	627(42.5%)	73(24.0%)
	Labour work	748(50.7%)	167(54.9%)
	Never worked	101(6.8%)	64(21.1%)
Lifestyles			
Hazardous drinking	No	1405(97.0%)	284(96.6%)
	Yes	44(3.0%)	10(3.4%)
Ever smoked	No	1133(76.8%)	248(80.8%)
	Yes	343(23.2%)	59(19.2%)
Being physically active	Not at all/not very active	329(22.3%)	231(75.7%)
	Fairly/Very active	1146(77.7%)	74(24.3%)
Any walks of half a kilometre or more in the last month	No	288(19.5%)	229(74.6%)
	Yes	1186(80.5%)	78(25.4%)
Frequency of eating fish	Never	66(4.5%)	33(10.9%)
	Some days	680(46.1%)	145(47.7%)
	Most days	544(36.9%)	94(30.9%)
	Every day	186(12.6%)	32(10.5%)
Frequency of eating fruits and vegetables	<2 servings/day	402(27.3%)	111(38.5%)
	2–4 servings/day	990(67.2%)	168(58.3%)
	>4 servings/day	82(5.6%)	9(3.1%)
Physical health			
Hearing impairment	No	1201(81.4%)	165(53.9%)
	Yes	275(18.6%)	141(46.1%)
Vision impairment	No	847(57.4%)	165(54.3%)
	Yes	629(42.6%)	139(45.7%)
Traumatic Brain Injury	No	1428(96.7%)	280(91.5%)
	Yes	48(3.3%)	26(8.5%)
High blood pressure	No	578(39.2%)	95(31.1%)
	Yes	897(60.8%)	210(68.9%)
Diabetes	No	960(65.0%)	183(59.8%)
	Yes	516(35.0%)	123(40.2%)
Heart problems	No	1212(82.2%)	222(73.8%)
	Yes	263(17.8%)	79(26.2%)
Stroke	No	1400(94.9%)	251(81.8%)
	Yes	76(5.1%)	56(18.2%)
Transient Ischemic Attack	No	1435(97.2%)	280(92.1%)
	Yes	41(2.8%)	24(7.9%)
Obesity	No	965(83.8%)	74(88.1%)
	Yes	186(16.2%)	10(11.9%)
Sleep health			
Sleep problems	No	1278(86.9%)	208(68.6%)
	Yes	193(13.1%)	95(31.4%)
Daytime sleepiness	No	1431(97.4%)	153(88.4%)
	Yes	38(2.6%)	20(11.6%)
Mental health			
Depressive symptoms	No	1222(82.8%)	222(72.1%)
	Yes	254(17.2%)	86(27.9%)
Objective social connections			
Currently married	No	521(35.3%)	198(64.3%)
	Yes	954(64.7%)	110(35.7%)
Living alone	No	1361(92.2%)	254(82.5%)
	Yes	115(7.8%)	54(17.5%)
Frequency of meeting families	Monthly or less	234(15.9%)	55(18.0%)
	Weekly or more	1241(84.1%)	251(82.0%)
Frequency of meeting friends	Monthly or less	672(45.6%)	246(80.1%)
	Weekly or more	803(54.4%)	61(19.9%)
Frequency of meeting neighbours	Monthly or less	507(34.3%)	205(67.0%)
	Weekly or more	969(65.7%)	101(33.0%)
Number of friends	None	487(33.0%)	206(69.4%)
	One-two	293(19.9%)	47(15.8%)
	Three-five	457(31.0%)	39(13.1%)
	Six and above	238(16.1%)	5(1.7%)
Number of neighbours	None	315(21.4%)	158(53.6%)
	One-two	564(38.4%)	105(35.6%)
	Three-five	459(31.2%)	30(10.2%)
	Six and above	131(8.9%)	2(0.7%)
Attending religious activities	No	511(34.6%)	232(75.3%)
	Yes	965(65.4%)	76(24.7%)
Attending community meetings or social clubs	No	1156(78.3%)	286(93.2%)
	Yes	320(21.7%)	21(6.8%)
Subjective social connections			
Feeling lonely	No	1298(88.4%)	131(78.4%)
	Yes	170(11.6%)	36(21.6%)
Satisfaction with friend support in neighbourhood	No	435(30.3%)	190(71.7%)
	Yes	1002(69.7%)	75(28.3%)
Difficulty in maintaining a friendship	No	1415(95.9%)	167(54.4%)
	Yes	61(4.1%)	140(45.6%)

### Network structure

**Fig. 1:**
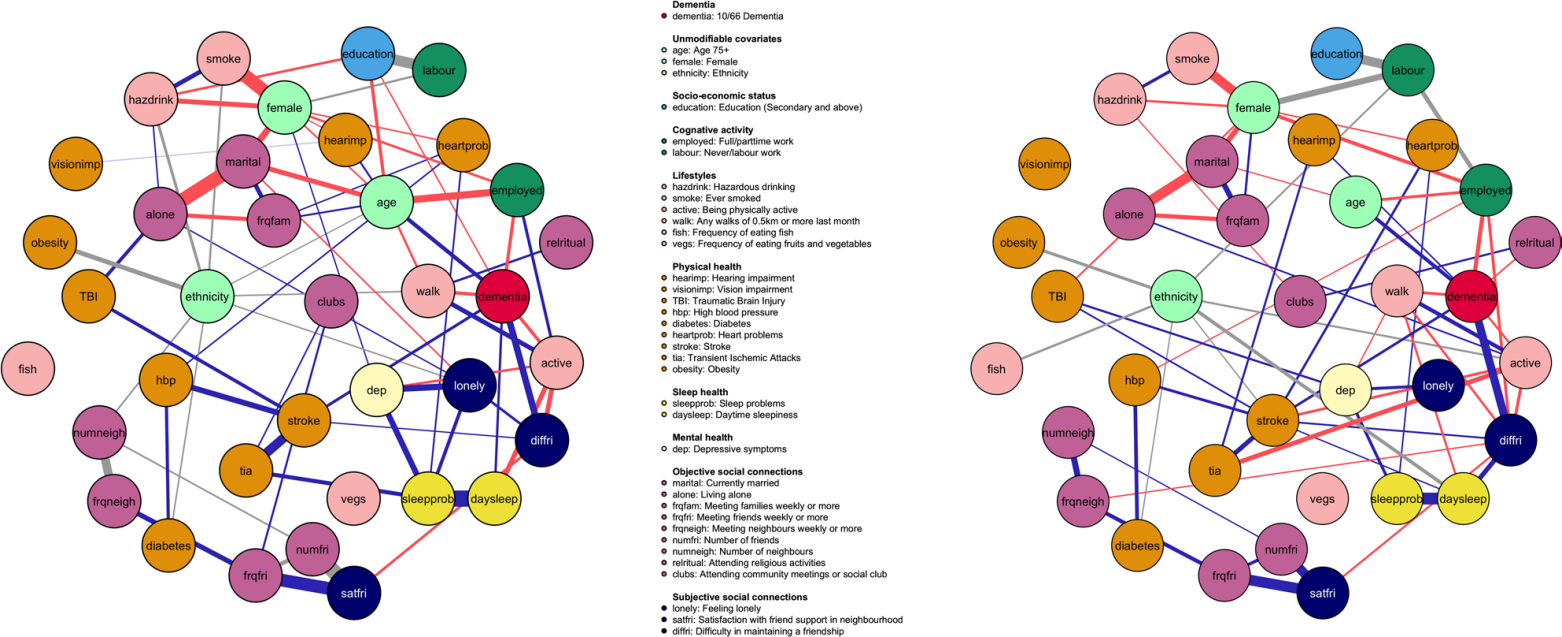
Network structure of dementia and its associated factors in WiSE 2023 (left) and WiSE 2013 (right). Network structure was estimated by mixed graphical modelling using cross-validation alongside the “AND” rule, to tune the parameter in L1-penalization. Each domain of factors is represented by colors. Each edge corresponds to the conditional independence between two nodes. The thickness of an edge represents the absolute weight of the connection (the thicker the edge, the stronger the connection), and only connections with weights larger than 0.22 are presented for clarity. The color of the edge represents the sign of the connection with blue for positive connections, red for negative connections and grey for undefined connections. The layout was plotted using the Fruchterman and Reingold layout for aesthetical reasons and was forced to be the same across waves for comparison

To avoid false positive results, only edge weights ≥0.22 are displayed in Fig. 1, and the network structure with all edges can be found in Supplementary Figure 2. Network structures were found to be similar across both datasets: 1) the correlation of edge weights between network structure in WiSE 2023 and that in WiSE 2013 was 0.8; 2) majority of the associations were not found to vary across two waves (see Supplementary Table 6 for the 17 connections found to vary across waves). Therefore, we report results from WiSE 2023 as the main results.

In WiSE 2023, 25 of all 37 factors were found to have a direct association with dementia in the network (Table 2). Factors that had an edge weight ≥0.22 with dementia were age over 75 years (edge weight=0.49), educational attainment of secondary or above (edge weight=−0.24), being employed (edge weight=−0.42), being physically active (edge weight=−0.43), any walks of half a kilometre or more in the last month (edge weight=−0.44), stroke (edge weight=0.38), daytime sleepiness (edge weight=0.34), and difficulty in maintaining a friendship (edge weight=0.76). As *mgm* fits a generalised linear model to estimate the edge weight and all variables in our network are dichotomised or categorical, the strength of the edge weight can be understood in terms of log odds.

**Table 2:** Variables that are directly associated with dementia and bootstrap results^a^

**Node names**	**Node labels**	**Edge weight**	**Bootstrapped mean** **(95% CI)**
diffri	Difficulty in maintaining a friendship	0.76	0.90 (0.52,1.29)
age	Age 75+	0.49	0.56 (0.21,0.92)
walk	Any walks of 0.5km or more last month	−0.44	−0.46 (−0.79,−0.12)
active	Being physically active	−0.43	−0.47 (−0.86,0.00)
employed	Full/parttime work	−0.42	−0.58 (−1.18,0.00)
stroke	Stroke	0.38	0.41 (0.00,0.89)
daysleep	Daytime sleepiness	0.34	0.43 (0.00,1.07)
education	Education (Secondary or above)	−0.24	−0.26 (−0.63,0.00)
frqfri	Meeting friends weekly or more	−0.21	−0.30 (−0.76,0.00)
relritual	Attending religious activities	−0.14	−0.18 (−0.50,0.00)
clubs	Attending community meetings or social club	−0.12	−0.21 (−0.71,0.00)
numfri^b^	Number of friends	0.11	0.28 (0.00,0.61)
female	Female	0.1	0.17 (0.00,0.60)
marital	Currently married	0.04	−0.16 (−0.55,0.00)
visionimp	Vision impairment	0.03	−0.16 (−0.48,0.00)
vegs^b^	Frequency of eating fruits and vegetables	0.03	0.11 (0.00,0.51)
labour	Never/labour work	0.02	0.09 (0.00,0.33)
hearimp	Hearing impairment	0.02	0.06 (0.00,0.35)
lonely	Feeling lonely	0.02	−0.14 (−0.58,0.00)
TBI	Traumatic Brain Injury	0.01	0.16 (0.00,0.76)
heartprob	Heart problems	0.01	0.03 (−0.18,0.33)
obesity	Obesity	0.01	−0.01 (−0.37,0.35)
hazdrink	Hazardous drinking	0.01	0.09 (−0.22,0.69)
frqneigh	Meeting neighbours weekly or more	0.01	−0.07 (−0.40,0.00)
numneigh^b^	Number of neighbours	0.01	0.11 (0.00,0.37)

^a^The CI of edge-weights was calculated using the non-parametric bootstrap with wider 95% CI indicating more unstable estimate. The bootstrapped 95% CI of edge-weights cannot be used to test for significance of an edge being different from zero for LASSO regularized edges

^b^Sign direction for categorical variables were undefined by default thus presented as positive values during bootstrapping stage

**Fig. 2:**
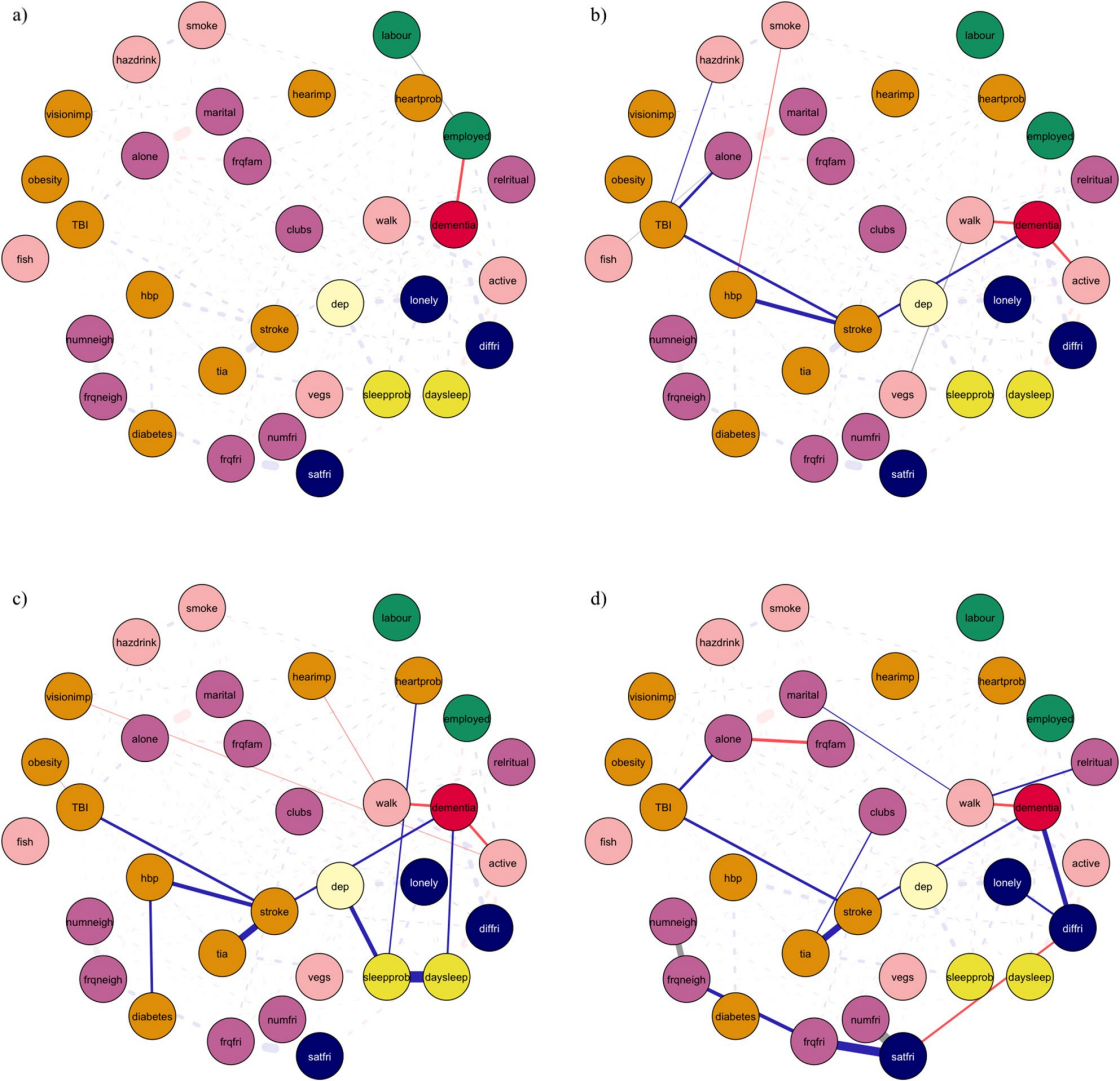
Shortest paths from each domain of associated factors to dementia in WiSE 2023. **a** cognitive activity; **b** lifestyles; **c** physical, mental and sleep health; **d** objective and subjective social connections

The shortest path from each factor to dementia was via the above-identified factors with direct and strong associations with dementia. Being female was connected to dementia via a lower likelihood of being employed, and ethnicity was connected to dementia via any walks of half a kilometre or more in the last month. The shortest path from the remaining factors to dementia is displayed in Fig. 2. In general, lifestyle was connected to dementia via blood pressure or TBI and stroke. Physical and mental health were connected to dementia via different paths: vision and hearing impairments via being physically inactive and no walks of half a kilometre or more in the last month respectively; TBI, TIA and high blood pressure via stroke; and depressive symptoms and heart problems via sleep health. The shortest paths from social connections to dementia varied by the specific aspects of social connections: less frequent interaction with families via living alone, TBI, and stroke; less frequent interaction with friends and neighbourhood via dissatisfaction with friend support in the neighbourhood and difficulty in maintaining a friendship; and feeling lonely via difficulty in maintaining a friendship.

### Centrality of nodes

The top 10 variables that were highly connected to other variables in the network were gender, age, dementia, sleep problems, currently married, living alone, stroke, being physically active, satisfaction with support from friends in the neighbourhood, and ethnicity (Supplementary Figure 3b). In contrast, several chronic conditions (e.g. diabetes, vision impairment, obesity and hearing problems) and lifestyle variables (e.g. diet) were peripheral in the network, suggesting weaker or fewer direct associations with other variables once all interrelations were considered. However, several network variables, particularly those reflecting social connections, are conceptually related and correlated (e.g., marital status with living alone, sleep problems with daytime sleepiness, satisfaction with support from friends in the neighbourhood with frequency of meeting friends and number of friends). Therefore, high strength centrality of these variables may reflect strong within-domain associations rather than broader relevance across domains.

### Stability and accuracy of the network

Supplementary Figure 3a depicts the stability of the edge weights of all possible connections. The sizable bootstrapped CIs indicate that many edge weights likely did not differ from one another (Supplementary Figure 4). Noticeably, the connections of age, any walks of half a kilometre or more in the last month, and difficulty in maintaining a friendship with dementia were shown to be reliably strong and stable.

The case-dropping bootstrap examining stability of node strength returns a CS-coefficient of 0.55, which means that a maximum of 55% of the participants can be dropped while the node strength would retain, with 95% certainty, a correlation higher than 0.7 with the original node strength (see Supplementary Figure 3c).

### Sensitivity analysis

Across sensitivity analyses, a largely consistent set of factors showed direct associations with dementia (edge weight ≥0.22). Age, being employed, stroke, being physically active, any walks of half a kilometre or more in the last month, and difficulty in maintaining a friendship were identified as directly and strongly associated with dementia in at least five of the six sensitivity analyses. Daytime sleepiness was also repeatedly detected, appearing in three of the four network models in which this variable was retained (Supplementary Figure 5 and Supplementary Table 7).

A stable set of nodes also ranked among the top 10 variables with the highest strength across sensitivity analyses, although the exact ordering varied (Supplementary Table 7). Age, gender, dementia, stroke, and being physically active consistently appeared among the top 10 most central nodes in all sensitivity analyses, while social and sleep-related variables (e.g., marital status, living alone, sleep problems, and daytime sleepiness) were frequently identified.

## Discussion

Using two nationally representative cross-sectional studies of older adults in Singapore, this study applied network analysis to map the interrelations among sociodemographic, behavioural, physical, mental, and social factors in relation to dementia status. We identified a set of highly interconnected variables, underscoring the complexity of dementia and its multifactorial nature. Age, active employment, physical activity, social connections, sleep health and stroke were detected to have strong and direction connections with dementia, consistently across surveys, gender and ethnic groups, suggesting robust cross-cutting patterns within the Singaporean ageing population. These nodes also had stronger inter-connections with other variables within the networks, while other physical conditions such as obesity, diabetes, heart problems, vision and hearing impairment, and behavioural factors such as diet appeared more peripheral within the network. This peripheral positioning should not be interpreted as diminished etiological importance, particularly given the cross-sectional design and evidence that many dementia risk factors exert their effects earlier in the life course or indirectly through more proximal factors [6, 24].

While causality cannot be inferred from cross-sectional data, our findings may have practical relevance for dementia prevention and care strategies that acknowledge the interconnected nature of late-life factors. The strong connections of age, active employment, physical activity, social connections with other variables in the network highlight how socio-demographic, behavioural, and social contexts are intertwined with dementia and other late-life health domains. This pattern reinforces the potential value of population-level initiatives that encourage continued engagement in work and community activities, promote physical functioning, and foster social connectedness throughout later life [40].

Given the cross-sectional design of this study, the observed associations of active employment, physical activity and social connections with dementia are likely to reflect bidirectional or reciprocal processes rather than unidirectional causal pathways. On the one hand, continued engagement in work may serve as a marker of preserved cognitive functioning, which is associated with lower risk of dementia independent of physical and social functioning [41–43]. Similarly, higher levels of physical activity have been associated with reduced risk of dementia, potentially through mechanisms related to cerebrovascular health, inflammation, and neuroplasticity [24]. Greater social engagement and stronger social networks have likewise been linked to lower risk of dementia risk, possibly by providing cognitive stimulation, buffering stress, and supporting emotional well-being [24]. On the other hand, cognitive impairment may limit occupational participation, physical capacity, and social functioning, leading to workforce exit, sedentary lifestyles and social withdrawal among individuals with dementia [44, 45]. Taken together, the clustering of employment, physical activity, and social connections within dementia-related networks underscores their central role within the broader system of late-life cognitive health.

Within the networks, sleep problems and stroke were directly associated with dementia with an edge strength comparable to the above-mentioned key factors, ranking among the most connected nodes and lying on the shortest paths from many factors to dementia. Their positions within the network suggest that these conditions frequently coexist with dementia and with each other. Given their bidirectional relationships with cognitive function [46–48], promoting sleep health and cerebrovascular disease prevention could yield broad benefits across ageing-related domains among individuals with and without dementia.

Some well-established factors from other domains, such as lifestyle factors (excluding physical activity), and physical and mental health conditions, displayed weaker or no associations with dementia in these cross-sectional networks. Several explanations are plausible. First, reverse causality may attenuate observed links, as individuals often modify their health behaviours (e.g. smoking or hazardous drinking) following diagnosis [49, 50]. Second, physiological changes in late life may obscure associations that are evident in midlife [51–53], such as weight loss or blood pressure decline preceding dementia onset [54, 55]. Third, these well-established factors may function as distal factors which may operate indirectly through more proximal factors, as captured within the network structure. For example, hazardous drinking may relate to dementia primarily through TBI and stroke [56, 57], while sensory impairments may connect to dementia via reduced physical activity. However, longitudinal studies are warranted to further validate these potential pathways.

### Policy implications

Although we did not evaluate specific interventions and causal inference cannot be drawn from cross-sectional network analysis, the observed direct and strong associations of employment, physical activity, and social connections with dementia in the network mirror several core pillars of Singapore’s Action Plan for Successful Ageing, including re-employment, volunteerism, communities of care, and intergenerational engagement in later life [58]. Thus, policies promoting continued engagement in work, maintenance of physical function, and social participation may be relevant components of broader dementia prevention and care strategies.

Beyond aligning with existing policy priorities, the network structure also points to potential directions for integrated, multi-domain programmes. For instance, the observed pathways linking sleep disturbance, cerebrovascular risk, and dementia highlight the potential value of community-based sleep health initiatives combined with vascular risk screening. Similarly, the prominence of social connectivity within the network underscores the potential benefit of strengthening opportunities for social participation through existing community and volunteer platforms.

Importantly, despite Singapore's multi-ethnic composition, we did not observe substantial differences in the associations between key factors and dementia across ethnic groups. This suggests that the interconnected relationships linking employment, physical activity, social connections, and health-related factors to dementia are broadly shared across the population, supporting a whole-population rather than ethnicity-specific approach. From a policy perspective, this reinforces the value of inclusive initiatives that promote healthy ageing across all older adults, while remaining sensitive to cultural diversity in modes of participation. Rather than tailoring interventions by ethnicity, population-wide programmes that offer flexible pathways for engagement, such as community activities, volunteering opportunities, and age-friendly employment, may be better positioned to leverage common protective structures identified in the network.

Our study has several limitations. First, the cross-sectional nature of the data precludes establishing temporal precedence, such that neither the directionality of associations nor the ordering of direct and indirect pathways can be determined, rendering the observed associations susceptible to reverse causality. Nonetheless, the primary aim of this study was not to infer causality, but to characterise the pattern of conditional interrelations among dementia and its established associated factors in a complex system. Second, our study did not collect data on all established factors, including exposure to air pollution and pesticides, and hypercholesterolemia. Third, some measurements may lack comprehensive construct validity. For example, diet was assessed via frequency of fish and vegetables/fruits intake, and cognitive activity was proxied using employment status and the highest job level one ever had. However, these proxies are commonly used, and expected associations were largely observed. Finally, we did not differentiate dementia subtypes, for which network structures may differ [43].

Nonetheless, our study has numerous strengths. It provides novel insights into the complex interactions among dementia and its diverse factors, using two nationally representative samples collected a decade apart with consistent methodology, and the results were robust to a comprehensive series of sensitivity analyses. Our study, encompassing ethnic groups of Chinese, Malaysian, and Indian descent, provides relevance and implications to populations in China, Malaysia, and India. Additionally, it demonstrates the utility of network analysis in public health, covering common methodological challenges such as missing data, sampling weighting, and categorical variables.

## Conclusions

Our study mapped the complex web of interconnections among dementia and its associated factors in two large samples of older adults in Singapore. The networks revealed consistent clustering of demographic, behavioural, and health domains, with age, physical activity, social connections, sleep health and stroke occupying central positions. These findings provide a data-driven representation of how multiple factors coexist in later life, offering a foundation for longitudinal investigations and integrative care strategies that recognise dementia as embedded within a broader system of ageing-related interdependencies rather than as a condition driven by single causes.

## Supplementary Information

Supplementary Material 1.

## Data Availability

The datasets generated and/or analysed during the current study are not publicly available due to ethical requirements but are available from the corresponding author on reasonable request.

## Abbreviations

LIBRA: Lifestyle for BRAin health
CAIDE: Cardiovascular Risk Factors, Aging and Incidence of Dementia
ANU-ADRI: Australian National University Alzheimer’s Disease Risk Index
WHO: World Health Organization
WiSE: Well-being of the Singapore Elderly study
TBI: traumatic brain injury
TIA: transient ischemic attack
GMS: Geriatric Mental State
AGECAT: Automated Geriatric Examination of Computer Assisted Taxonomy
CSI'D: Community Screening Instrument for Dementia
CERAD: Consortium to Establish a Registry for Alzheimer’s Dementia
COGSCORE: global cognitive score of participants
RELSCORE: informant score reflecting the informant’s observations of cognitive and functional decline in the respondent
DSM-IV: Diagnostic and Statistical Manual of Mental Disorders Fourth Edition
MGM: mixed graphical model
MI: multiple imputation
CI: confidence interval
DAG: Directed Acyclic Graph
