# Supplementary material for: Mapping the network structure of dementia and its associated factors among older adults in Singapore: evidence from two national cross-sectional studies
Source: BMC Geriatr. 2026 May 13;26:915. doi: 10.1186/s12877-026-07535-2 (PMC13339258; doi:10.1186/s12877-026-07535-2)
Supplement: Supplementary file 1 — Supplementary Material 1. [file 12877_2026_7535_MOESM1_ESM.docx]

**A network approach to dementia: pathways among dementia and its determinants**

**Supplementary Materials**

[Supplementary Table 1. Study procedures and key considerations 2](#_Toc221193582)

[Supplementary Table 2. Well established determinants and their operationalization in WiSE datasets 1](#_Toc221193583)

[Supplementary Table 3. Fit indices of for latent class model of household wealth and distribution of household indices across classes 4](#_Toc221193584)

[Supplementary Table 4. Differences between complete cases and non-complete cases 5](#_Toc221193585)

[Supplementary Table 5. Missing pattern of all variables 7](#_Toc221193586)

[Supplementary Text 1. Multiple Imputation 8](#_Toc221193587)

[Supplementary Text 2. Rules of defining sign direction for categorical variables 8](#_Toc221193588)

[Supplementary Figure 1. Network structure estimated using Extended Bayesian Information Criterion (EBIC) 9](#_Toc221193589)

[Supplementary Figure 2. The undirected network structure of dementia and its determinants in WiSE 2023 and WiSE 2013. 10](#_Toc221193590)

[Supplementary Table 6. Associations that vary by wave, ethnicity and sex 11](#_Toc221193591)

[Supplementary Figure 3. Accuracy of undirected network structure. 12](#_Toc221193592)

[Supplementary Figure 4. Bootstrapped difference test between connections of interest. 13](#_Toc221193593)

[Supplementary Figure 5. Other sensitivity analyses. 14](#_Toc221193594)

[Supplementary Table 7. Factors with direct association with dementia (>=0.22) and top 10 factors with highest strength centrality for each sensitivity analysis 15](#_Toc221193595)

[Reference 15](#_Toc221193596)

# Supplementary Table 1. Study procedures and key considerations

| Study Procedure | Key considerations |
| --- | --- |
| Step 1:  Selection of key determinants | - Inclusion: well-established determinants identified in at least one of the 2020 and 2024 reports of the Lancet Commission,^1,2^ the WHO guidelines,^3^ and popular dementia index (LIBRA,^4^ CAIDE,^5^ ANU-ADRI^6^) - Exclusion: Not available in our datasets |
| Step 2:  Variable operationalisation | - See Supplementary Table 2&3 - Variables are dichotomized where possible or treated as unordered categorical variables because Mixed Graphical Modelling (MGM) cannot deal with ordered categorical variables and does not define sign for unordered categorical variable^7^. |
| Step 3:  Sampling weights | - As disproportionate stratified sampling was applied to oversample older adults and minorities, sampling weights were applied to restore population representativeness. In WiSE 2013, the target proportion were 26% for adults aged 75-84 years and 22% for those aged ≥85 years, whereas in WiSE 2023, the corresponding targets were 34% and 31%, respectively. For both survey waves, the target was 30% for both Malay and Indian participants. - Sampling weights were calculated based on census population by June 2022 for WiSE 2023 and by June 2011 of WiSE 2013 excluding those whose ethnicity are Others (source: <https://www.tablebuilder.singstat.gov.sg/table/TS/M810671>). - When estimating the network structure, sampling weights were incorporated via the built-in argument for weights (“weights”) of the *mgm* package in R. - During the bootstrapping procedure to check accuracy and stability of the network, sampling weights were incorporated via the custom estimation function in *bootnet* package in R. Basically, participants were first sampled with equal probability and then assigned their sampling weights during the estimation procedure. |
| Step 4:  Multiple Imputation | - Participants with complete data differed significantly from those with missing data, see Supplementary Table 4, thus multiple imputation using the mice package (version 3.16.0) was applied.^9^ - Refer to Supplementary Text 1 for more details |
| Step 5:  Network estimation and calculation of shortest paths between nodes of interest | - MGM was chosen as it can deal with network of categorical variables (binary or nominal) with different levels and incorporate sampling weights.^7^ - Cross-validation (CV) was used to select the tuning parameter controlling the L1-penalization (a.k.a LASSO) instead of Extended Bayesian Information Criterion (EBIC) as the latter produces unreasonably sparse network (Supplementary Figure 1). Meanwhile, as CV tends to be less conservative than EBIC,^7^ to avoid false positive results, we only presented edges with a weight ≥0.22 for clarity (exponentiate of ±0.22 is 0.8/1.25, which is approximately the minimal clinically important differences utilised by the guidelines for the National Institute for Health and Clinical Excellence (NICE) in the UK.^10^ - We used the "AND" rule to combine estimates from neighbourhood regression, which requires all estimates of pairwise interactions to be nonzero to set the edge to be present. - Network structure and edge strength/sign were averaged across imputed datasets (Supplementary Text 2) - Shortest path from determinants in domains of cognitive activity, lifestyle, physical, mental and social health was calculated by omitting age, gender, ethnicity and education attainment.^11^ - Node strength was calculated to assess the centrality of a node, rather than closeness and betweenness given their challenging interpretation as centrality indices in psychological networks.^12^ |
| Step 6:  Check network accuracy | - MI Boot (pooled sample) procedure to construct valid bootstrapped CIs when multiple imputation was adopted to address missing data.^13^ (100 bootstrapped samples were drawn for each imputed dataset^14^). Bootstrapping for each imputed sample was done using the *bootnet* package (version 1.6),^15^ and the estimates were then stacked to get the 95% bootstrapped CI. - Accuracy of network ^14^ - accuracy of edge weights was examined by constructing 95% bootstrapped CIs using non-parametric bootstrap - stability of node strength was examined by calculating correlation-stability coefficient (the maximum proportion of cases that can be dropped to retain, with 95% certainty, a correlation with the original centrality of higher than 0.7) using the case-dropping subset bootstrap - differences between edge-weights of interest were tested by applying bootstrapped difference tests |
| Step 7:  Moderated network analysis | - Conduct moderated network analysis to investigate whether each pairwise association between variables in the network varied by wave, ethnicity and gender |
| Step 7:  Sensitivity analyses | - Recode variables as nominal variables instead of dichotomizing the variable to reserve more information (refer to Supplementary Text 2 for more details) - Incorporate emerging determinants into the network to investigate robustness of the network, including personal income, household wealth, number of life deficits, tooth loss and anxiety^4,16^ - Omit nodes with disproportional missingness among participants with and without dementia, including obesity (22.0% among non-dementia and 72.7% among dementia), daytime sleepiness (0.5% among non-dementia and 43.8% among dementia), feeling lonely (0.5% among non-dementia and 45.8% among dementia), and satisfaction with friend support in the neighbourhood (2.6% among non-dementia and 14.0% among dementia) - Omit nodes with direct and strong association with dementia which were also highly correlated with other variables (>0.8) to avoid the issue of random selecting highly correlated factors by LASSO^17^ - Apply "Multiple Imputation, Then Deletion" method to delete imputed diagnosis of dementia^18^ - Exclude participants whose information on socio-demographic and risk factors were provided by informants |

# Supplementary Table 2. Well established determinants and their operationalization in WiSE datasets

| Source | Variable | Questions | Operationalization |
| --- | --- | --- | --- |
|  | **Unmodifiable** |  |  |
|  | Age |  | Range 57-100 |
|  | Gender |  | 1=Female; 0=Male |
|  | Ethnicity |  | 1=Chinese; 2=Malay; 3=Indian |
|  | **Socio-economic status** |  |  |
| Lancet commission, CAIDE, ANU-ADRI | Educational attainment | What is your highest education level? | 0=None/Below primary/Primary; 1=Secondary or above |
|  | Home ownership | Does the family own or rent this home? | 1=Yes; 0=No |
|  | Number of rooms in the household | How many rooms are there? | 1=Two; 2=Three; 3=Four; 4=Five; 5=Six and above |
|  | Vehicle ownership | How many motor vehicles (motor bike, cars, vans or trucks) do family members own? | 1=Yes; 0=No |
|  | Number of household appliances | In this household is there: A television set? A fridge and/ or freezer? A telephone / mobile phone? Air Conditioning? Washing machine? Cable TV/ Paid TV channels? Personal computer or Internet subscription/access? | 1=0-4 appliances;  0=5 or more appliances |
|  | Personal income(SG$) | Do you receive any income, benefits, pensions or allowances? If Yes, please list any benefits or allowances with the approximate monthly income from each  Benefit type (Pension; CPF; Disability Benefit; Money from Family; Income from Rented Land or Property; Income from Paid Work; Public Assistance; Eldercare Benefits; Other, specify; No further benefits to enter) | 0=below 1000;  1=1000 and above; |
|  | Number of lifetime deficits | Where were you born? (City/Town, Village)  Can you read a newspaper?  Could you write a letter, if you needed to?  Do you ever go hungry because there is not enough food to eat?(Never, Some days/Most days/Every day) | 0=None;  1=One;  2=Two;  3=Three or more |
| LIBRA, ANU-ADRI, WHO | **Cognitive activity** |  |  |
|  | Being employed | Do you have a job? | 1=Full/part time job; 0= Unemployed/household wife/husband/Retired |
|  | Highest job level | What is the highest level job you have ever had?  What kind of work did you do in this job? | 1=Associate professional (eg technical, nursing, artistic)/Clerical worker /secretary/Manager/administrator/Professional (eg health, teaching, legal, financial)  2=Shop keeper/Skilled labourer (e.g building, electrical etc.)/Semi-skilled labourer (e.g helper of skilled labourer)/Unskilled labourer/Agricultural worker;  3=Never worked; |
|  | **Lifestyle** |  |  |
| Lancet commission, LIBRA, ANU-ADRI, WHO | Hazardous drinking | Before you reached the age of 60, what was the most you would drink in an average week? (Record maximum regular consumption in UNITS of alcohol per week)  What about after the age of 60? | 1=≥14 drinks per week for men and ≥7 drinks per week for women;  0=<14 drinks per week for men and <7 drinks per week for women |
| Lancet commission, LIBRA, ANU-ADRI, WHO | Ever smoked | Has there ever been a period when you smoked cigarettes, cigars, or a pipe, chewing tobacco, beedi or snuff nearly every day?  Do you still use tobacco regularly? | 0=Never smoked;  1=Past/current smoker; |
| LIBRA, ANU-ADRI, WHO | Frequency of eating fruits and vegetables | How many servings of fruit and vegetables have you eaten over the last 3 days?  (One fruit or one portion of salad or vegetables counts as a serving.)  How often do you eat fish? | 0=<2 servings/day; 1=2-4 servings/day; 2=>4 servings/day  0=Never; 1=Some days; 2=Most days; 3=Every day |
| Lancet commission, LIBRA, CAIDE, ANU-ADRI, WHO | Physical activity | In the last month, how many times did you do any walks  of half a kilometre or more?  Taking into account both work and leisure, would you say that you are? | 0=Never; 1=Walk at least once  0=Not at all/not very active;  1=Fairly/Very active; |
|  | **Physical Health** |  |  |
| Lancet commission, LIBRA | Hearing impairment | I'm going to read out a list of health problems, and for each one I'd like you to tell me please whether or not you generally have that problem at the present time--- Hearing difficulty or deafness | 1=Yes; 0=No |
| Lancet commission | Eyesight problems | I'm going to read out a list of health problems, and for each one I'd like you to tell me please whether or not you generally have that problem at the present time--- Eyesight problems | 1=Yes; 0=No |
|  | Tooth loss | Do you have any missing teeth? | 1=Yes; 0=No |
| Lancet commission, ANU-ADRI | Traumatic Brain Injury | Have you ever had a serious head injury in which you were knocked out?  Supplement information from:  179.3 Dysphasia (due to brain damage) words are muddled up or used incorrectly.  179.4 Dysarthria (due to brain damage). The interviewee has difficulty articulating words, but knows what he wants to say.  Did he/she ever have an accident resulting in a serious injury to his/her head or brain? | 1=Yes; 0=No |
| LIBRA, WHO | Heart problems | Have you ever been told by a doctor that you had heart trouble? | 1=Yes; 0=No |
| Lancet commission considered it as causes of dementia thus did not include as risk factors; LIBRA considered it not readily modifiable at an individual level thus did not include it in the index | Stroke | Have you ever had a stroke that needed medical attention. What happened at that time?  (Code only if clear history of sudden onset of unilateral paralysis, and/or loss of speech, and/or blindness lasting for at least 2 days) | 1=Yes; 0=No |
|  | Transient Ischemic Attack | Have you ever developed sudden weakness of a limb, loss of speech, or partial blindness which got better quickly, in less than one day? (Doctors sometimes call these attacks Transient Ischemic Attacks). | 1=Yes; 0=No |
| Lancet commission, LIBRA, CAIDE, WHO | High blood pressure | Self-reported diagnosis:  Have you ever been told by a doctor that you had high blood pressure?  Supplement information from:  I'm going to read out a list of health problems, and for each one I'd like you to tell me please whether or not you generally have that problem at the present time--- High blood pressure  Has a doctor ever told him/her that he/she had raised blood pressure?(from informant) | 0=No doctor-diagnosed high blood pressure;  1=Has doctor-diagnosed high blood pressure; |
| Lancet commission, LIBRA, ANU-ADRI, WHO | Diabetes | Have you ever been told by a doctor that you had diabetes?  Do you need a special diet, take tablets, or have insulin injections? | 0=No doctor-diagnosed diabetes;  1=Has doctor-diagnosed diabetes; |
| Lancet commission, LIBRA, CAIDE, ANU-ADRI, WHO | Obesity | BMI≥30 kg/m2 | 1=Yes; 0=No |
| LIBRA | Sleep problems | Have you had trouble sleeping or change pattern recently?  Did he/she have difficulty sleeping? (from informants)  Has he/she had particular difficulty sleeping? (from informants) | 1=Yes; 0=No |
| LIBRA | Daytime sleepiness | Has insomnia for most of the night and sleeps mainly during the day?  Supplemented by interviewer observation:  Patient repeatedly falls asleep and has to be awakened. | 1=Yes; 0=No |
|  | **Mental health** |  |  |
| Lancet commission, LIBRA, ANU-ADRI, WHO | Depression | Depression section of Geriatric Mental State AGECAT | 0=Normal;  1=Depressive symptoms |
|  | Anxiety | Anxiety section of Geriatric Mental State AGECAT | 0=Normal;  1=Anxiety symptoms |
| Lancet commission, ANU-ADRI, WHO, LIBRA | **Social connections** |  |  |
|  | Marital status | Are you currently married? | 0=Never married/Widowed/Divorced/Separated  1=Married/Co-habiting; |
|  | Living alone | Records of coresidents | 1=Yes; 0=No |
|  | Frequency of meeting children/relatives | How often do you see any of your children or other relatives to speak to? | 0=Monthly or less;  1=Weekly or more; |
|  | Frequency of meeting friends | Do you have friends in this neighbourhood/estate?  How often do you have a chat or do something with one of your friends? | 0=Monthly or less;  1=Weekly or more; |
|  | Frequency of meeting neighbours | How often do you see any of your neighbours to have a chat or do something with? | 0=Monthly or less;  1=Weekly or more; |
|  | Number of friends | Do you have friends in this neighbourhood/estate?  Which close friends do you meet or contact regularly (at least once a month)? | 0=None;  1=One to two;  2=Three to five;  3=Six or above; |
|  | Number of neighbours | How many good neighbours do you have whom you meet or talk to regularly (at least once a month)? | 0=None;  1=One to two;  2=Three to five;  3=Six or above; |
|  | Religious activities | Do you attend religious meetings/ visit religious places? | 0=No; 1=Yes; |
|  | Attending community meetings or social club | Do you attend meetings of any community or social groups, such as clubs, lectures or anything like that? | 0=No; 1=Yes; |
|  | Feeling lonely | Do you feel lonely? | 1=Yes; 0=No |
|  | Satisfaction with friend | All in all are you satisfied or dissatisfied with the help and support you can get from your close friends? | 0=No friend/not satisfied;  1=Satisfied; |
|  | Social difficulties | How much difficulty did you have in maintaining a friendship? | 1=Mild/Moderate/Severe/Extreme difficulty;  0=None |
|  | **Not available** |  |  |
| LIBRA, CAIDE, ANU-ADRI, WHO, Lancet commission | Hypercholesterolemia | Not Available |  |
| Lancet commission | Air pollution | Not Available |  |
| ANU-ADRI | Pesticide exposure | Not Available |  |
| LIBRA | Renal dysfunction | Not Available |  |

1) Lancet commission = 12 modifiable factors identified in "Livingston G, Huntley J, Sommerlad A, et al. Dementia prevention, intervention, and care: 2020 report of the Lancet Commission. Lancet 2020; 396: 413–46." and "Livingston G, Huntley J, Liu KY, et al. Dementia prevention, intervention, and care: 2024 report of the Lancet standing Commission. Lancet 2024; 404: 572–628.";

2) LIBRA (LIfestyle for BRAin health) = 15 modifiable factors identified in "Rosenau C, Köhler S, Soons LM, et al. Umbrella review and Delphi study on modifiable factors for dementia risk reduction. Alzheimers Dement 2024; 20: 2223–39.";

3) CAIDE (the Cardiovascular Risk Factors, Aging, and Incidence of Dementia) = 7 factors (5 modifiable factors in addition to age and sex) identified in "Kivipelto M, Helkala EL, Laakso MP, et al. Midlife vascular risk factors and Alzheimer’s disease in later life: longitudinal, population based study. BMJ 2001; 322: 1447–51. ";

4) ANU-ADRI (the Australian National University Alzheimer’s Disease Risk Index) = 15 factors (13 modifiable factors in addition to age and sex) identified in "Anstey KJ, Cherbuin N, Herath PM. Development of a new method for assessing global risk of Alzheimer’s disease for use in population health approaches to prevention. Prev Sci 2013; 14: 411–21.";

5) WHO = 12 modifiable factors identified in "World Health Organization. Risk reduction of cognitive decline and dementia: WHO guidelines. Geneva: World Health Organization, 2019 https://iris.who.int/handle/10665/312180 (accessed May 14, 2024)."

# Supplementary Table 3. Fit indices of for latent class model of household wealth and distribution of household indices across classes

To reduce number of nodes in the network, we conducted latent class analysis to derive classes of participants reflecting household wealth using four variables (house ownership, vehicle ownership, number of rooms in the household and household appliance insufficiency). Three-class solution was selected based on AIC, BIC and proportion of each class and defined as deprived, adequate and affluent based on the distribution of household indices.

Supplementary Table 2.1 Fit indices of for latent class model of household wealth

|  | AIC | BIC | Classes |
| --- | --- | --- | --- |
| 1 class | 9707 | 9746 |  |
| 2 classes | 8871 | 8954 | Class1: 8.2%  Class2: 91.8% |
| 3 classes | 8649 | 8777 | Class1: 7.5%  Class2: 53.8%  Class3: 38.7% |
| 4 classes (not converge) | 8657 | 8830 | Class1: 7.4%  Class2: 24.4%  Class3: 17.7%  Class4: 50.4% |
| 5 classes (not converge) | 8659 | 8876 | Class1: 18.1%  Class2: 3.4%  Class3: 4.3%  Class4: 25.8%  Class5: 48.4% |

Supplementary Table 2.2 Distribution of household index across three-class solution

|  | Deprived | Adequate | Affluent |
| --- | --- | --- | --- |
| House ownership (Yes) | 7.4% | 99.5% | 99.1% |
| Vehicle ownership (Yes) | 8.0% | 4.2% | 51.5% |
| Number of rooms in household |  |  |  |
| Two | 53.7% | 0.0% | 0.1% |
| Three | 43.9% | 9.8% | 1.1% |
| Four | 1.2% | 41.9% | 5.6% |
| Five | 1.3% | 46.5% | 51.5% |
| Six and above | 0.0% | 1.8% | 41.8% |
| Household appliance insufficiency (Yes) | 48.0% | 11.4% | 0.4% |

# Supplementary Table 4. Differences between complete cases and non-complete cases

| Variable labels | level | Frequency (%) | Missing (%) | OR (95% CI) |
| --- | --- | --- | --- | --- |
| Age | 60-74 | 1088(54.6%) | 0% | Ref |
|  | 75 and above | 905(45.4%) |  | **2.88(2.40, 3.46)** |
| Gender | Male | 900(45.2%) | 0% | Ref |
|  | Female | 1093(54.8%) |  | **1.46(1.22, 1.74)** |
| Ethnicity | Chinese | 678(34.0%) | 0% | Ref |
|  | Malay | 699(35.1%) |  | **1.45(1.17, 1.79)** |
|  | Indian | 616(30.9%) |  | 0.99(0.79, 1.24) |
| Education | Primary or below | 1170(58.9%) | 0.4% | Ref |
|  | Secondary or above | 816(41.1%) |  | **0.45(0.37, 0.54)** |
| Personal income | Less than SG$1000 | 1185(59.8%) | 0.5% | Ref |
|  | SG$1000 or above | 798(40.2%) |  | **0.37(0.31, 0.45)** |
| Household wealth | Deprived | 139(7.2%) | 3.0% | Ref |
|  | Adequate | 1013(52.4%) |  | **0.68(0.48, 0.97)** |
|  | Affluent | 781(40.4%) |  | **0.41(0.29, 0.60)** |
| Number of lifetime deficits | None | 642(32.9%) | 2.1% | Ref |
|  | 1 disadvantage | 799(40.9%) |  | **1.32(1.06, 1.64)** |
|  | 2 disadvantages | 286(14.7%) |  | **2.39(1.79, 3.18)** |
|  | 3 or 4 disadvantages | 225(11.5%) |  | **5.64(4.03, 7.89)** |
| Dementia | No | 1476(82.7%) | 10.5% | Ref |
|  | Yes | 308(17.3%) |  | **10.50(7.78, 14.17)** |
| Being employed | No | 1355(68.2%) | 0.3% | Ref |
|  | Yes | 632(31.8%) |  | **0.44(0.36, 0.54)** |
| Highest job level | Manager/Professional | 797(40.2%) | 0.5% | Ref |
|  | Labourer | 1017(51.3%) |  | **1.84(1.52, 2.23)** |
|  | Never worked | 169(8.5%) |  | **3.30(2.34, 4.66)** |
| Hearing impairment | No | 1533(77.2%) | 0.4% | Ref |
|  | Yes | 452(22.8%) |  | **1.72(1.39, 2.12)** |
| Eyesight problems | No | 1116(56.3%) | 0.5% | Ref |
|  | Yes | 867(43.7%) |  | **1.20(1.00, 1.43)** |
| Tooth loss | No | 240(13.1%) | 8.3% | Ref |
|  | Yes | 1587(86.9%) |  | **1.54(1.15, 2.08)** |
| Traumatic Brain Injury | No | 1903(95.9%) | 0.4% | Ref |
|  | Yes | 82(4.1%) |  | 1.17(0.75, 1.83) |
| High blood pressure | No | 765(38.6%) | 0.5% | Ref |
|  | Yes | 1218(61.4%) |  | 1.13(0.94, 1.36) |
| Diabetes | No | 1287(64.8%) | 0.4% | Ref |
|  | Yes | 698(35.2%) |  | 1.08(0.90, 1.30) |
| Heart problems | No | 1603(81.0%) | 0.7% | Ref |
|  | Yes | 376(19.0%) |  | 1.14(0.91, 1.43) |
| Stroke | No | 1846(93.0%) | 0.4% | Ref |
|  | Yes | 140(7.0%) |  | **1.88(1.33, 2.66)** |
| Transient Ischemic Attacks | No | 1912(96.4%) | 0.5% | Ref |
|  | Yes | 71(3.6%) |  | 1.33(0.83, 2.13) |
| Obesity | No | 1169(84.9%) | 30.9% | Ref |
|  | Yes | 208(15.1%) |  | **0.52(0.33, 0.84)** |
| Sleep problems | No | 1658(83.7%) | 0.6% | Ref |
|  | Yes | 323(16.3%) |  | **2.08(1.63, 2.65)** |
| Daytime sleepiness | No | 1784(96.5%) | 7.2% | Ref |
|  | Yes | 65(3.5%) |  | **2.33(1.41, 3.86)** |
| Depressive symptoms | No | 1609(80.7%) | 0.0% | Ref |
|  | Yes | 384(19.3%) |  | **1.57(1.26, 1.96)** |
| Anxious symptoms | No | 1603(80.4%) | 0.0% | Ref |
|  | Yes | 390(19.6%) |  | 1.23(0.99, 1.54) |
| Ever hazardous drinking | No | 1875(96.5%) | 2.5% | Ref |
|  | Yes | 69(3.5%) |  | 1.24(0.76, 2.00) |
| Ever smoke | No | 1534(77.2%) | 0.4% | Ref |
|  | Yes | 452(22.8%) |  | 0.89(0.72, 1.11) |
| Frequency of eating fish | Never | 115(5.8%) | 0.5% | Ref |
|  | Some days | 915(46.1%) |  | **0.65(0.44, 0.96)** |
|  | Most days | 714(36.0%) |  | 0.68(0.46, 1.01) |
|  | Every day | 239(12.1%) |  | **0.48(0.31, 0.75)** |
| Frequency of eating fruit and vegetables | <2 servings/day | 574(29.2%) | 1.5% | Ref |
|  | 2-4 servings/day | 1286(65.5%) |  | 0.93(0.76, 1.13) |
|  | >4 servings/day | 104(5.3%) |  | 0.75(0.48, 1.15) |
| Being physically active | No | 607(30.6%) | 0.5% | Ref |
|  | Yes | 1376(69.4%) |  | **0.34(0.28, 0.41)** |
| Any walks of half a kilometre or more | Never | 549(27.7%) | 0.5% | Ref |
|  | At least once last month | 1434(72.3%) |  | **0.31(0.25, 0.38)** |
| Currently married | No | 829(41.7%) | 0.2% | Ref |
|  | Yes | 1160(58.3%) |  | **0.41(0.34, 0.49)** |
| Living alone | No | 1770(88.8%) | 0% | Ref |
|  | Yes | 223(11.2%) |  | **2.61(1.96, 3.49)** |
| Meeting families | Monthly or less | 364(18.3%) | 0.3% | Ref |
|  | Weekly or more | 1623(81.7%) |  | **0.68(0.54, 0.85)** |
| Meeting friends | Monthly or less | 1018(51.2%) | 0.3% | Ref |
|  | Weekly or more | 969(48.8%) |  | **0.56(0.47, 0.67)** |
| Meeting neighbours | Monthly or less | 799(40.2%) | 0.3% | Ref |
|  | Weekly or more | 1188(59.8%) |  | **0.50(0.42, 0.60)** |
| Number of friends | None | 764(38.6%) | 0.8% | Ref |
|  | One-two | 390(19.7%) |  | **0.55(0.43, 0.70)** |
|  | Three-five | 558(28.2%) |  | **0.43(0.34, 0.54)** |
|  | Six and above | 265(13.4%) |  | **0.33(0.24, 0.44)** |
| Number of neighbours | None | 529(26.9%) | 1.3% | Ref |
|  | One-two | 744(37.8%) |  | **0.50(0.40, 0.62)** |
|  | Three-five | 554(28.2%) |  | **0.38(0.29, 0.48)** |
|  | Six and above | 141(7.2%) |  | **0.24(0.16, 0.37)** |
| Attending religious activities | No | 817(41.1%) | 0.2% | Ref |
|  | Yes | 1173(58.9%) |  | **0.45(0.38, 0.54)** |
| Attending community meetings or social club | No | 1607(80.8%) | 0.2% | Ref |
|  | Yes | 382(19.2%) |  | **0.58(0.46, 0.73)** |
| Feeling lonely | No | 1597(86.7%) | 7.6% | Ref |
|  | Yes | 244(13.3%) |  | **1.79(1.37, 2.35)** |
| Satisfaction with friend support | No | 690(36.2%) | 4.4% | Ref |
|  | Yes | 1215(63.8%) |  | **0.43(0.35, 0.52)** |
| Difficulty in maintaining a friendship | No | 1775(89.4%) | 0.4% | Ref |
|  | Yes | 211(10.6%) |  | **4.46(3.23, 6.15)** |

# Supplementary Table 5. Missing pattern of all variables

| Number of variables with missing values | Number of participants |
| --- | --- |
| 0 | 1140 |
| 1 | 572 |
| 2 | 105 |
| 3 | 21 |
| 4 | 57 |
| 5 | 58 |
| 6 | 18 |
| 7 | 8 |
| 8 | 4 |
| 9 | 1 |
| 11 | 2 |
| 21 | 2 |
| 23 | 1 |
| 31 | 1 |
| 32 | 1 |
| 35 | 2 |
| Total | 1993 |

# Supplementary Text 1. Multiple Imputation

As shown in Supplementary Table 4, there were differences in many variables among complete participants (n=1140) and non-complete participants (n=853). Complete participants are those who have no missingness in all variables used in the network analysis. The proportion of missingness of each variable was below 10% apart from missingness of obesity status (30.9% in WiSE 2023) and dementia status (10.5% in WiSE 2023). Thus, multiple imputation by chained equations were performed to restore representativeness of our sample. All variables in the network analysis were included in the imputation stage ^20^.

The edge weight of each interaction was the average of the edge weight of the corresponding interaction over all the imputed datasets. For sign directions of an interaction, if opposite directions were detected across imputations, the direction was recoded as undefine (edge in grey colour); if no opposite directions, the direction is defined as the direction that is detected over 50% of the imputations among which the interaction was detected, otherwise the direction is left as undefined.

# Supplementary Text 2. Rules of defining sign direction for categorical variables

We attempted to retain the categorical form of a variable instead of dichotomising it, to capture the strength averaged over the rest of the levels of the categorical variable relative to the reference level, which is not affected by the distribution of the categorical variables. However, currently the *mgm* package for conducting mixed graphical modelling does not define signs for unorder categorical variables, we did not include this set of results as main results, and instead conducted it as a sensitivity check and included the details in this supplementary file.

In undirected networks estimated by the *mgm* package in R, an edge weight is the arithmetic mean of parameter estimates of each interaction. In other words, for a pairwise interaction between node s and r, the edge weight is the mean of absolute value of all parameters obtained from the regression of s on r and from the regression of r on s. In our model, we combine parameters using the AND-rule and set overparameterize to FALSE. This means for node s with m levels and node r with n levels, there are m*(n-1) parameters (set A) in the regression on s and n*(m-1) parameters (set B) in the regression on r, and the edge weight is the arithmetic mean of m*(n-1) and n*(m-1) parameters if not all parameters are zeros in both parameter sets across imputations. To further define sign direction between categorical variables, the two sets of parameter matrix were transformed and transposed to (m-1)*(n-1) by omitting the first row of parameters after subtracting them from each row in the parameter set (the subtraction will render the first row to be all zeros and thus can be removed). After getting two sets of (m-1)*(n-1) parameter matrix, the sign direction will be defined as positive if all the elements in the two parameter matrices are nonnegative and as negative if all the elements in the two parameter matrices are nonpositive, otherwise the sign direction will be left as undefined. The rule of combing sign direction of categorical variables across imputations is the same as described in Supplementary Text 4.


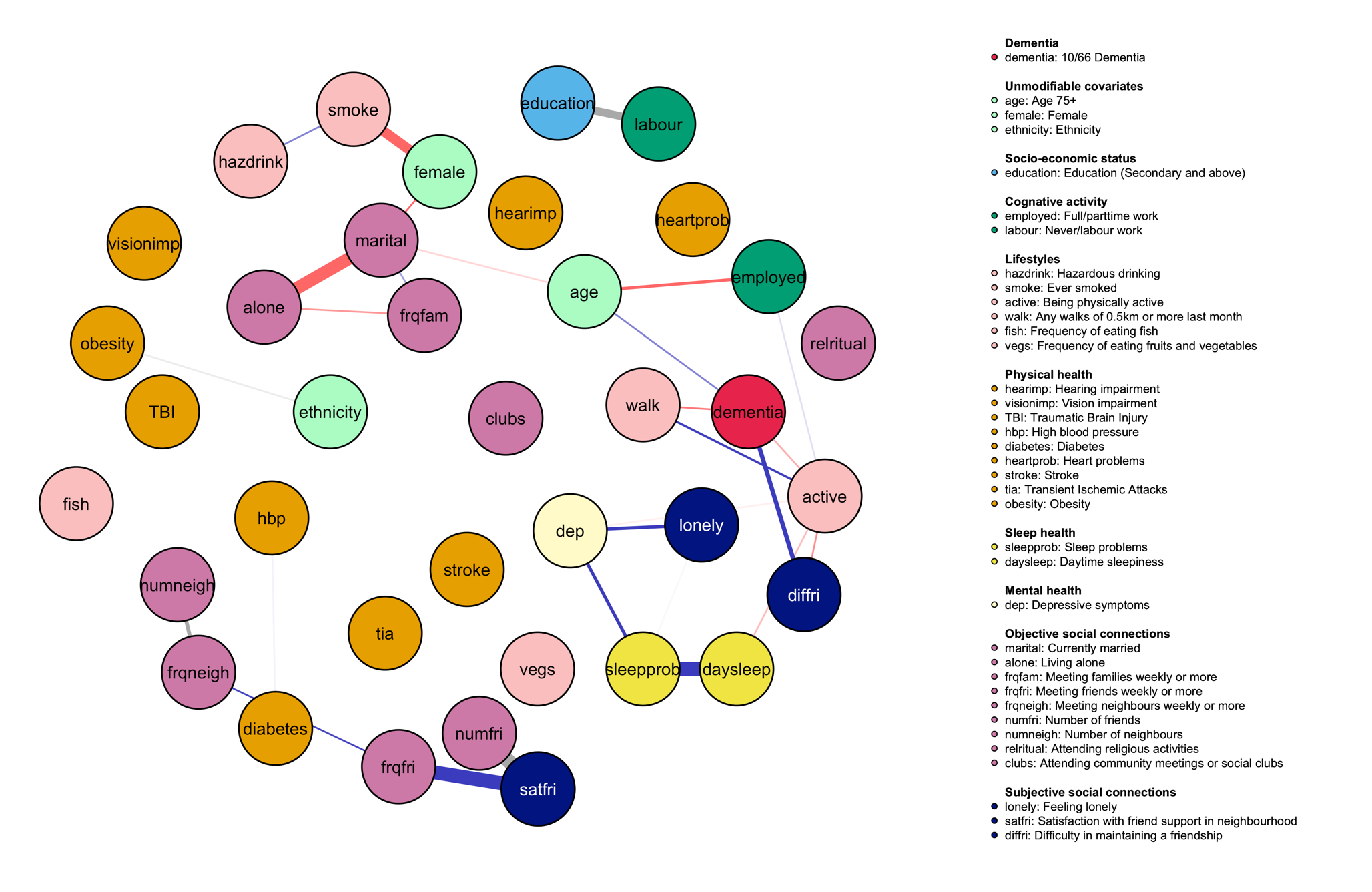


Supplementary Figure 1. Network structure estimated using Extended Bayesian Information Criterion (EBIC)


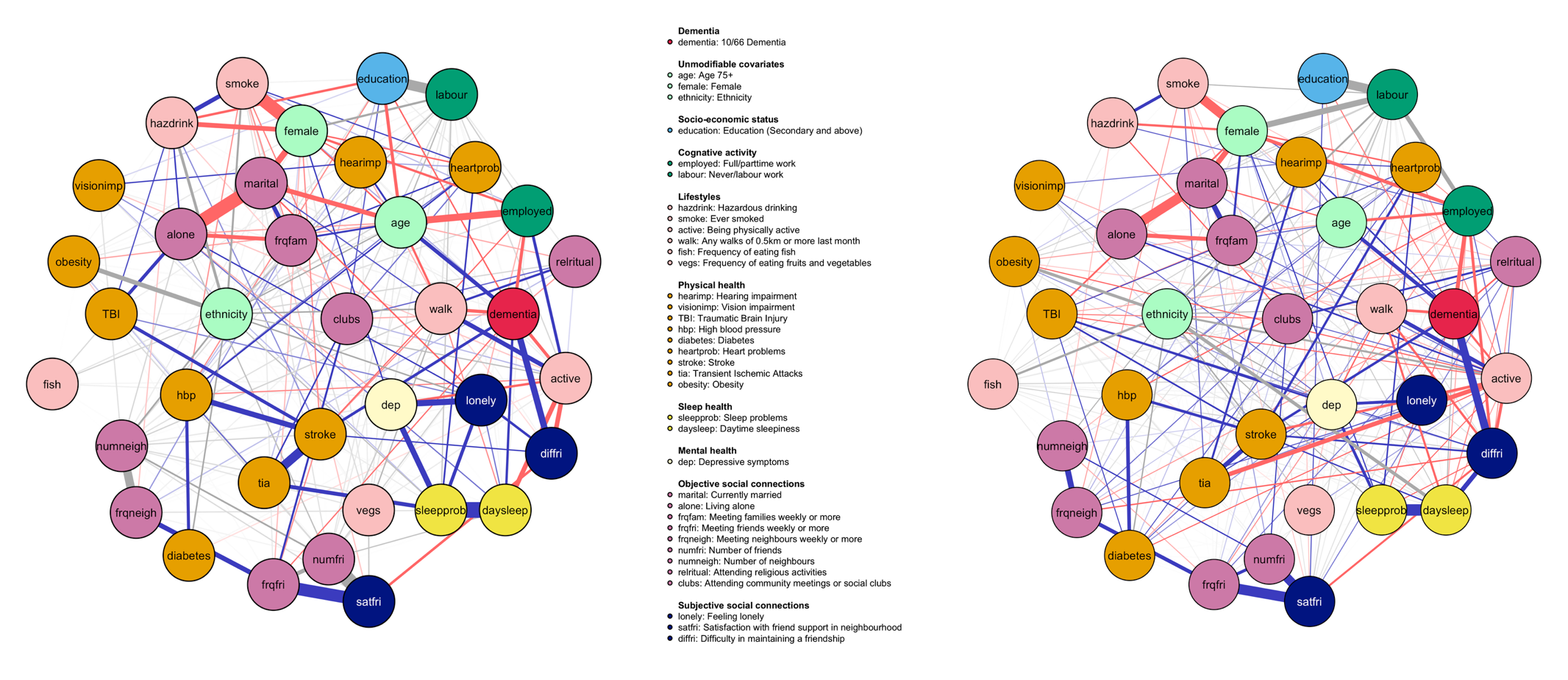


Supplementary Figure 2. The undirected network structure of dementia and its determinants in WiSE 2023 and WiSE 2013. Network structure was estimated by mixed graphical modelling using cross-validation alongside the “AND” rule, to tune the parameter in L1-penalization. Each domain of determinants is represented by colours. Each edge corresponds to the conditional independence between two nodes. The thickness of an edge represents the absolute weight of the connection (the thicker the edge, the stronger the connection). The colour of the edge represents the sign of the connection with blue for positive connections, red for negative connections and grey for undefined connections. The layout was plotted using the Fruchterman and Reingold layout for aesthetical reasons and was forced to be the same for comparison.

# Supplementary Table 6. Associations that vary by wave, ethnicity and gender

| By wave | By ethnicity | By gender |
| --- | --- | --- |
| ethnicity--dep | stroke--alone | hazdrink--clubs |
| TBI--dep | education--labour | age--marital |
| hazdrink--alone | labour--clubs | labour--frqfam |
| labour--clubs | female--smoke | numfri--numneigh |
| age--ethnicity | age--fish |  |
| lonely--diffri | age--education |  |
| ethnicity--hazdrink | age--TBI |  |
| hbp--numfri | female--employed |  |
| TBI--numfri | labour--fish |  |
| walk--numneigh | frqfam--fish |  |
| numfri--numneigh | numneigh--fish |  |
| ethnicity--active | diabetes--numneigh |  |
| female--smoke | employed--TBI |  |
| ethnicity--stroke | labour--tia |  |
| female--stroke |  |  |
| labour--TBI |  |  |
| numfri--vegs |  |  |

b)

a)


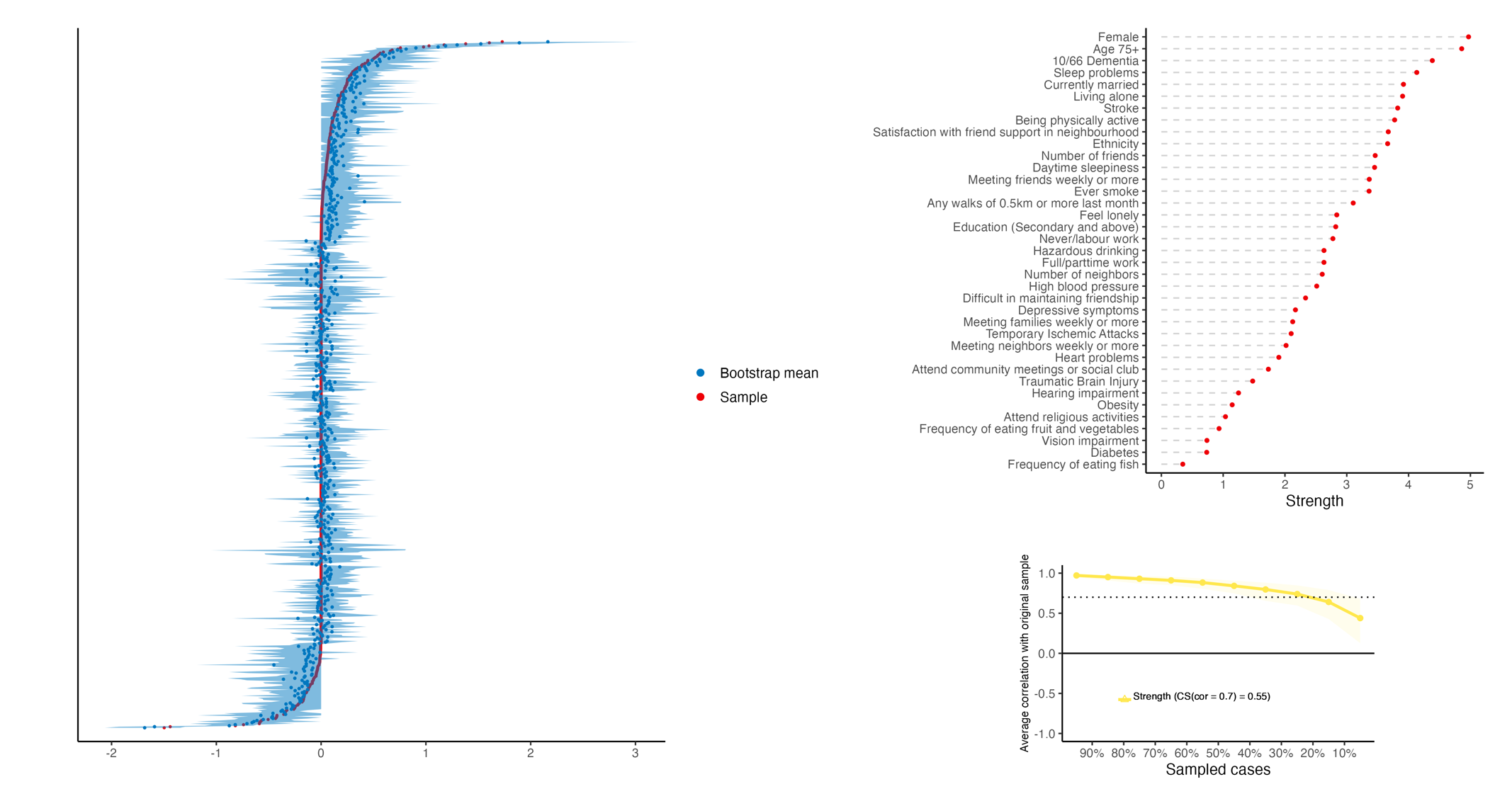


c)

Supplementary Figure 3. Accuracy of undirected network structure. a) Accuracy of edge weights based on 2000 nonparametric bootstrapped samples with red dots representing weights of original sample and blue dots representing mean weights of bootstrapped samples surrounded by the 95% bootstrapped confidence interval. A narrower confidence interval indicates good stability. b) Node strength in the estimated network. c) The correlation stability coefficient of node strength, the CS(cor=0.7), estimated using case-dropping subset bootstrap.


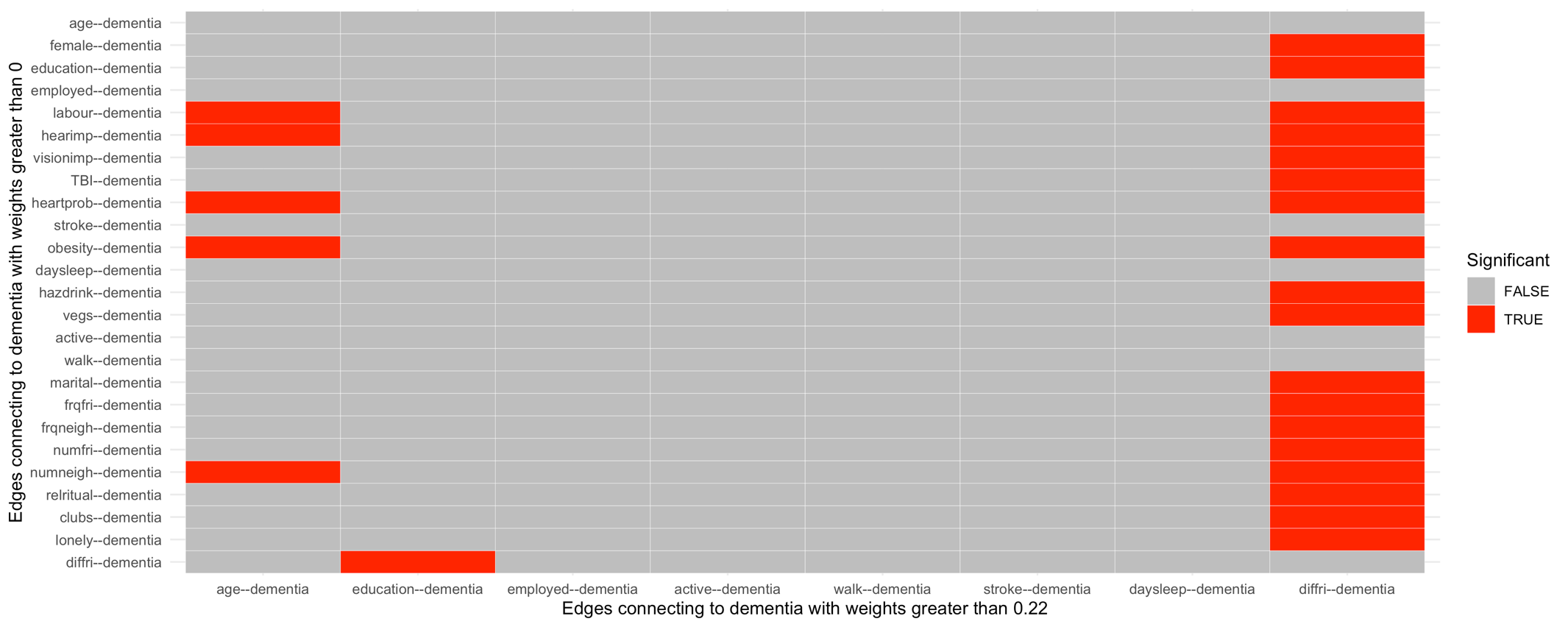


Supplementary Figure 4. Bootstrapped difference test between connections of interest. X-axis are connections with dementia that have an edge weight greater than 0.22, and y-axis are connections with dementia that have an edge weight greater than 0.


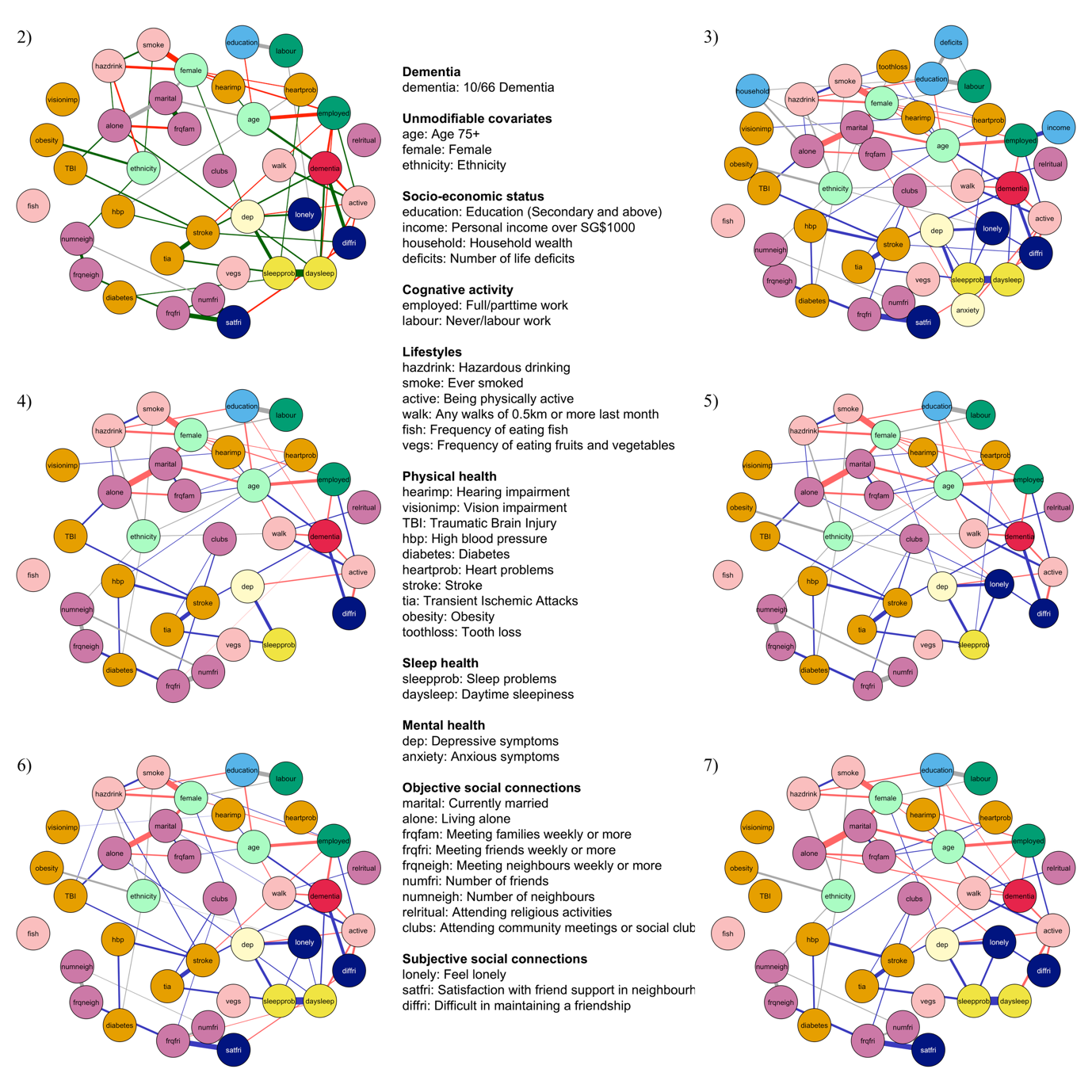


Supplementary Figure 5. Other sensitivity analyses. 2) recoding variables into categorical; 3) adding emerging determinants (i.e. income, household, deficits, toothloss, anxiety); 4) omitting nodes with disproportional missingness among dementia and non-dementia participants (i.e. obesity, daysleep, lonely, satfri); 5) omitting nodes with potential collinearity problems (i.e. daysleep, satfri); 6) the "multiple imputation, then deletion" (MID) method excluding participants with missing data on diagnosis of dementia; 7) excluding participants with information supplemented by informants

# Supplementary Table 7. Factors with direct association with dementia (>=0.22) and top 10 factors with highest strength centrality for each sensitivity analysis

|  | Sensitivity2 | Sensitivity3 | Sensitivity4 | Sensitivity5 | Sensitivity6 | Sensitivity7 |
| --- | --- | --- | --- | --- | --- | --- |
| Associated factors |  |  |  |  |  |  |
| 1 | age | age | age | age | age | age |
| 2 | employed | employed | education | education | employed | education |
| 3 | stroke | stroke | employed | employed | stroke | employed |
| 4 | daysleep | daysleep | stroke | stroke | daysleep | stroke |
| 5 | active | active | active | active | active | active |
| 6 | diffri | walk | walk | walk | walk | walk |
| 7 |  | diffri | frqfri | diffri | diffri | alone |
| 8 |  |  | diffri |  |  |  |
| Strength centrality |  |  |  |  |  |  |
| 1 | female | age | female | female | female | female |
| 2 | daysleep | female | age | age | age | age |
| 3 | dementia | sleepprob | dementia | dementia | dementia | sleepprob |
| 4 | age | stroke | active | alone | daysleep | marital |
| 5 | marital | alone | stroke | marital | sleepprob | active |
| 6 | satfri | dementia | marital | stroke | stroke | alone |
| 7 | sleepprob | ethnicity | alone | active | active | stroke |
| 8 | stroke | smoke | smoke | ethnicity | marital | ethnicity |
| 9 | active | active | walk | smoke | smoke | frqfri |
| 10 | ethnicity | employed | numfri | numfri | satfri | numfri |

# Reference

1 Livingston G, Huntley J, Sommerlad A, *et al.* Dementia prevention, intervention, and care: 2020 report of the Lancet Commission. *Lancet* 2020; **396**: 413–46.

2 Livingston G, Huntley J, Liu KY, *et al.* Dementia prevention, intervention, and care: 2024 report of the Lancet standing Commission. *Lancet* 2024; **404**: 572–628.

3 World Health Organization. Risk reduction of cognitive decline and dementia: WHO guidelines. Geneva: World Health Organization, 2019 https://iris.who.int/handle/10665/312180 (accessed May 14, 2024).

4 Rosenau C, Köhler S, Soons LM, *et al.* Umbrella review and Delphi study on modifiable factors for dementia risk reduction. *Alzheimers Dement* 2024; **20**: 2223–39.

5 Kivipelto M, Helkala EL, Laakso MP, *et al.* Midlife vascular risk factors and Alzheimer’s disease in later life: longitudinal, population based study. *BMJ* 2001; **322**: 1447–51.

6 Anstey KJ, Cherbuin N, Herath PM. Development of a new method for assessing global risk of Alzheimer’s disease for use in population health approaches to prevention. *Prev Sci* 2013; **14**: 411–21.

7 Haslbeck JMB, Waldorp LJ. mgm: Estimating time-varying mixed graphical models in high-dimensional data. *J Stat Softw* 2020; **93**: 1–46.

8 Briganti G, Scutari M, McNally RJ. A tutorial on Bayesian networks for psychopathology researchers. *Psychol Methods* 2022; **28**: 947–61.

9 White IR, Royston P, Wood AM. Multiple imputation using chained equations: Issues and guidance for practice. *Statist Med* 2011; **30**: 377–99.

10 National Institute for Health and Care Excellence. NICE guidance. 2025. https://www.nice.org.uk/guidance/conditions-and-diseases (accessed Feb 24, 2025).

11 Isvoranu A-M, van Borkulo CD, Boyette L-L, *et al.* A network approach to psychosis: Pathways between childhood trauma and psychotic symptoms. *Schizophr Bull* 2017; **43**: 187–96.

12 Bringmann LF, Elmer T, Epskamp S, *et al.* What do centrality measures measure in psychological networks? *J Abnorm Psychol* 2019; **128**: 892–903.

13 Schomaker M, Heumann C. Bootstrap inference when using multiple imputation. *Stat Med* 2018; **37**: 2252–66.

14 Epskamp S, Borsboom D, Fried EI. Estimating psychological networks and their accuracy: A tutorial paper. *Behav Res* 2018; **50**: 195–212.

15 Epskamp S, Fried EI. bootnet: Bootstrap methods for various network estimation routines. 2024; published online Feb 21. https://cran.r-project.org/web/packages/bootnet/index.html (accessed June 12, 2024).

16 Bransby L, Rosenich E, Maruff P, Lim YY. How modifiable are modifiable dementia risk factors? A framework for considering the modifiability of dementia risk factors. *J Prev Alzheimers Dis* 2024; **11**: 22–37.

17 Freijeiro-González L, Febrero-Bande M, González-Manteiga W. A critical review of LASSO and its derivatives for variable selection under dependence among covariates. *Int Stat Rev* 2022; **90**: 118–45.

18 Sullivan TR, Salter AB, Ryan P, Lee KJ. Bias and Precision of the “Multiple Imputation, Then Deletion” method for dealing with missing outcome data. *Am J Epidemiol* 2015; **182**: 528–34.

19 Du J, Boss J, Han P, *et al.* Variable selection with multiply-imputed datasets: choosing between stacked and grouped methods. *J Comput Graph Stat* 2022; **31**: 1063–75.

20 Mainzer RM, Nguyen CD, Carlin JB, Moreno-Betancur M, White IR, Lee KJ. A comparison of strategies for selecting auxiliary variables for multiple imputation. *Biometrical Journal* 2024; **66**: 2200291.

21 Scutari M, Silander T, Ness R. bnlearn: Bayesian network structure learning, parameter learning and inference. 2024; published online May 2. https://cran.r-project.org/web/packages/bnlearn/index.html (accessed June 12, 2024).
